# Prevalence and diversity of type VI secretion systems in a model beneficial symbiosis

**DOI:** 10.3389/fmicb.2022.988044

**Published:** 2022-09-14

**Authors:** Andrea M. Suria, Stephanie Smith, Lauren Speare, Yuzhou Chen, Iris Chien, Emily Grace Clark, Madelyn Krueger, Alexander M. Warwick, Hannah Wilkins, Alecia N. Septer

**Affiliations:** ^1^Department of Earth, Marine and Environmental Sciences, University of North Carolina at Chapel Hill, Chapel Hill, NC, United States; ^2^Department of Microbiology, Oregon State University, Corvallis, OR, United States

**Keywords:** type VI secretion system, *Euprymna scolopes*, competition, symbiosis, *Aliivibrio fischeri*, roseobacter

## Abstract

The type VI secretion system (T6SS) is widely distributed in diverse bacterial species and habitats where it is required for interbacterial competition and interactions with eukaryotic cells. Previous work described the role of a T6SS in the beneficial symbiont, *Vibrio fischeri*, during colonization of the light organ of *Euprymna scolopes* squid. However, the prevalence and diversity of T6SSs found within the distinct symbiotic structures of this model host have not yet been determined. Here, we analyzed 73 genomes of isolates from squid light organs and accessory nidamental glands (ANGs) and 178 reference genomes. We found that the majority of these bacterial symbionts encode diverse T6SSs from four distinct classes, and most share homology with T6SSs from more distantly related species, including pathogens of animals and humans. These findings indicate that T6SSs with shared evolutionary histories can be integrated into the cellular systems of host-associated bacteria with different effects on host health. Furthermore, we found that one T6SS in *V. fischeri* is located within a genomic island with high genomic plasticity. Five distinct genomic island genotypes were identified, suggesting this region encodes diverse functional potential that natural selection can act on. Finally, analysis of newly described T6SSs in roseobacter clade ANG isolates revealed a novel predicted protein that appears to be a fusion of the TssB-TssC sheath components. This work underscores the importance of studying T6SSs in diverse organisms and natural habitats to better understand how T6SSs promote the propagation of bacterial populations and impact host health.

## Introduction

Bacteria must compete for habitat space in both free-living surface-associated environments and within animal hosts. The type VI secretion system (T6SS) is a molecular syringe used by bacteria to inject effector proteins into neighboring prokaryotic and eukaryotic cells and, in most cases, mediates contact-dependent competition (Cianfanelli et al., 2016). T6SS gene clusters have been detected in diverse bacterial genomes from a wide range of habitats (Boyer et al., 2009). The activity of the T6SS was first characterized in *Pseudomonas aeruginosa* (Mougous et al., 2006) and *Vibrio cholerae* (Pukatzki et al., 2006), and its activity has since been functionally described in other pathogenic bacteria, such as *Escherichia coli* (Aschtgen et al., 2008), *Serratia marcescens* (Murdoch et al., 2011), *Acinetobacter baumannii* (Carruthers et al., 2013), *Vibrio parahaemolyticus* (Salomon et al., 2013), *Vibrio vulnificus* (Church et al., 2016), and many others (reviewed in Hood et al., 2017). The T6SS has also been characterized in symbiotic and environmental bacteria, including *Agrobacterium tumefaciens* (Wu et al., 2008), *Bacteroidetes* (Russell et al., 2014b), *Burkholderia thailandensis* (Schwarz et al., 2010b), and *Rhizobium leguminosarum* (Bladergroen et al., 2003). Many of these bacterial species encode multiple, evolutionarily distinct T6SS gene clusters that are often located on genomic islands (Boyer et al., 2009; Schwarz et al., 2010b). Five T6SS subtypes have been described to date (Boyer et al., 2009), which may have diversified as strains adapted to competition against other bacteria or eukaryotic cells in the environment (Schwarz et al., 2010a). While these 13 core structural T6SS genes are well conserved in *Proteobacteria*, there is a lot of variability in the type of effector/immunity genes and accessory genes found within the genomic islands containing T6SS clusters (Boyer et al., 2009).

The Hawaiian bobtail squid, *Euprymna scolopes*, provides a uniquely tractable model system to study the role of T6SS activity in a host because it possesses two symbiotic organs. This model has been best studied for its light organ symbiosis, a binary association with the bioluminescent bacterium, *Vibrio fischeri*, which provides camouflage for the host in the form of counterillumination (Nyholm and McFall-Ngai, 2021; Visick et al., 2021). Female *E. scolopes* also possess a second, consortial symbiosis in the accessory nidamental gland (ANG, Collins et al., 2012), where bacteria are housed before transfer to the jelly coat of the squid eggs when they are laid. This bacterial community is composed of *Alphaproteobacteria*, *Verrucomicrobia*, *Gammaproteobacteria*, and *Flavobacteriia* (Collins et al., 2012; Kerwin and Nyholm, 2017), and has been found to protect the eggs from biofouling during development (Kerwin et al., 2019). These symbiotic organs both contain areas where competition would naturally occur during colonization, namely in the crypts of the light organ and the tubules of the ANG. Symbionts grow to high cell densities in these structures, between 10^7^–10^8^ cells in an adult light organ (Ruby and Asato, 1993) and 10^9^–10^10^ cells in a mature ANG (Collins, 2014), which would allow symbionts to use contact-dependent competition mechanisms like the T6SS.

Previous work identified T6SS clusters in *V. fischeri* genomes from squid and fish light organs (Speare et al., 2018). It was found that *V. fischeri* possess one T6SS cluster on the first chromosome (T6SS1), while a subset of genomes encode a second cluster on the second chromosome (T6SS2). The T6SS2 is necessary for killing other *V. fischeri* strains *in vitro* and to compete for colonization space in the *E. scolopes* light organ crypts (Speare et al., 2018, 2022; Guckes et al., 2019). The role of the T6SS1 remains unknown. These previous studies determining strain-specific occurrence of T6SS2 only screened 33 isolates by PCR detection for the presence of the T6SS2 genes *tssM_2* (*icmF_2*) and *tssD_2* (*vasA_2*) and did not address whether there were other genes in the genomic island when T6SS2 was not detected.

Type VI secretion system-associated genes were previously reported in 12 ANG and egg jelly coat isolate genomes (Collins et al., 2015; Gromek et al., 2016; Suria et al., 2020), however the number of T6SS gene clusters, their diversity, and predicted effectors were not characterized. The majority of these genomes are from *Leisingera* and *Ruegeria* symbionts, which are members of the roseobacter clade of *Alphaproteobacteria*, the most abundant class of symbionts in the ANG (Collins et al., 2012) and an abundant and biogeochemically important group in free-living marine microbial communities (Buchan and Moran, 2005; Wagner-Döbler and Biebl, 2006). Activity of these T6SS clusters has not been experimentally verified. While T6SS gene clusters have been detected in other roseobacter clade genomes and metagenomes (Persson et al., 2009; Kempnich and Sison-Mangus, 2020), experimentally verified activity of any T6SS in this group has not been reported to date. Therefore, more information is needed about the prevalence and diversity of T6SSs in the ANG to generate testable hypotheses about their functional roles during symbiosis.

Here, we used multiple bioinformatic approaches to analyze the genomes of 57 squid/fish light organ symbionts, 15 squid ANG symbionts, and 175 bacterial reference genomes in order to: (1) identify T6SS gene clusters, (2) group T6SSs into phylogenetically-distinct subtypes, and (3) examine the gene content in the T6SS gene clusters and genomic islands. This analysis revealed that the majority of isolates encode at least one T6SS from one of four subtypes, yet the function of only one T6SS symbiont type (T6SS2 in *V. fischeri*) has been experimentally verified. Furthermore, we identified a new class of sheath protein, found only in certain roseobacter clade genomes, that appears to be a fusion of the large and small sheath subunits (TssBC). Our *in silico* analysis indicates that the squid model system, which houses a diversity of secretion systems and novel structural genes, provides an opportunity to further our understanding of the ecological roles and essential structural components of T6SSs in a natural, co-evolved system.

## Materials and methods

### Bacterial strain isolation

New *Vibrio fischeri* strains were isolated from the light organs of three adult Hawaiian bobtail squid, *Euprymna scolopes*, and from a light organ-like structure of one sub-adult Atlantic brief squid, *Lolliguncula brevis* (Supplementary Table S1). All *E. scolopes* samples were donated by the lab of Dr. Eric Stabb and originally collected from Maunalua Bay, Oahu, HI, maintained in aquaria at the University of Georgia, and flash frozen upon death. Frozen samples were transported on dry ice to the University of North Carolina at Chapel Hill and were stored at −80°C until dissection. The *L. brevis* sample was obtained from a trawl net in Morehead City, NC, United States and was immediately dissected.

All dissecting tools were sterilized with 70% ethanol prior to use. Frozen animals were thawed on ice at 4°C prior to dissection. Tissue samples were dissected and washed three times by brief vortexing in filter sterilized artificial seawater (FSASW) and transferred to fresh FSASW for each wash. Washed samples were homogenized in fresh FSASW, serially diluted on LBS (Luria-Bertani with added salts) agar (Stabb et al., 2001), and incubated aerobically at 24°C for 48 h. Single colonies were streaked for isolation.

### Genome sequencing and analysis

The 10 squid light organ isolates obtained in this study and one previously isolated Hawaiian seawater strain (Supplementary Table S2) were grown for 16.5 h in 3.0 ml LBS broth at 24°C, 200 rpm shaking. Genomic DNA was extracted from 1.0 ml of pelleted cells using the Zymo Quick-DNA Fungal/Bacterial kit (Irvine, CA, United States) following manufacturer protocols. Samples were lysed in the “ZR BashingBead Lysis Tubes” for 10 min using a Vortex-Genie 2. Concentration and purity of DNA were measured on a spectrophotometer (Eppendorf BioSpectrophotometer basic).

Sequencing libraries were prepared using the Illumina Nextera kit series and sequenced using paired end (2 × 150 bp) reads on the Illumina NextSeq 550 to a depth of 150 Mb at the Microbial Genome Sequencing Center (MiGS, Pittsburgh, PA, United States). An average of 1.4 ± 0.18 M raw, paired end reads were sequenced per sample.

Raw reads were trimmed using Trimmomatic v.0.36 (Bolger et al., 2014) with a sliding window size of 10, sliding window minimum quality of 20, leading/trailing minimum quality of 3, and minimum read length of 50. Quality trimmed reads were assembled using SPAdes v.3.13.0 with default settings (Bankevich et al., 2012). Genome completion estimates were determined using CheckM v.1.0.18 (Parks et al., 2015), which determines the number of single copy housekeeping genes present in a genome compared to a list of marker genes specific for that genome’s placement on a reference tree. Average nucleotide identity (ANI) was calculated using FastANI (Jain et al., 2018). The Whole Genome Shotgun project and raw reads are deposited at DDBJ/ENA/GenBank under the BioProject accession PRJNA785243.

### Phylogenetic analysis of T6SS gene clusters

Genomes from 57 squid/fish light organ symbionts, 15 accessory nidamental gland (ANG) symbionts, and 175 bacterial reference strains were analyzed for the presence and type of T6SS gene clusters (Supplementary Table S3). Genomes that were not sequenced in this study were obtained from Genbank. All genomes were annotated using Prokka v.1.12 with default settings (Seemann, 2014), and the python and R script HAMBURGER (HMmer Based UndeRstandinG of gene clustERs; https://github.com/djw533/hamburger; Mariano et al., 2019) was used to detect T6SS gene clusters. A T6SS gene cluster was predicted if it contained at least four of the 13 core T6SS genes (*tssA-M*) and any given core gene was separated by no more than 10 non-core genes in a cluster. These criteria would detect core T6SS clusters and likely exclude auxiliary clusters that may only contain one or two toxin/anti-toxin system effector genes (Dong et al., 2013). The HAMBURGER script uses MUSCLE v.3.8.21 (Edgar, 2004) and FastTree v.2.1.11 (Price et al., 2010) to build a maximum-likelihood tree from the aligned TssBC (small and large subunits of the contractile sheath) amino acid sequences. The R packages “gggenes” and “ggplot2” were used to draw T6SS operons. The pangenomics workflow of Anvi’o v.7 (Eren et al., 2015; Delmont et al., 2018) was used to compare the T6SS2 genomic region of *V. fischeri* genomes. This region is the area between the flanking genes *manA* (mannose-6-phosphate isomerase) and *tRNA-Gly* on the second chromosome. Any genomes that had only one break in the assembly in this region and could easily be concatenated were included. T6SS effectors were predicted by BLASTP search against the experimentally validated effector protein SecReT6 database (Li et al., 2015), downloaded on January 28, 2022. Results were considered significant if the amino acid similarity was ≥30% and E-value ≤10^−6^.

### Coincubation assay

The nine *V. fischeri* strains isolated from *E. scolopes* in this study (Supplementary Table S2) were screened for the ability to inhibit the growth of *V. fischeri* ES114 pVSV102 on agar plates as described previously (Speare et al., 2018). All strains were grown overnight at 24°C on LBS agar with the addition of 100 μg/ml kanamycin for maintaining the pVSV102 plasmid with GFP fluorescence. Cells from these plates were resuspended in 0.5 ml LBS broth, washed once with LBS, and normalized to an OD600 of 1.0. Each strain was mixed with *V. fischeri* ES114 pVSV102 in a 1:1 ratio and 10 μl of the mixture was spotted on LBS agar. These coincubation spots were incubated for 24 h at 24°C and then imaged for GFP fluorescence to determine the extent to which *V. fischeri* ES114 pVSV102 was able to grow or be inhibited in the presence of control and test strains. Representative images are shown from three experimental trials, where each trial contained three resuspensions per strain, prepared from different colonies.

## Results

### Assembly and species identification of new vibrio genomes

To add to the diversity of *Vibrio fischeri* genomes available for T6SS analysis in squid light organs, we sequenced the genomes of nine *V. fischeri* strains from three *E. scolopes* light organs and one *V. fischeri* strain from a light organ-like structure in *L. brevis.* All genomes were assembled to 99.9% completion with genome sizes of 4.0–4.4 Mbp (Supplementary Table S2). All strains were assigned to the species *V. fischeri* based on ANI analysis with a cutoff of ≥95% (Supplementary Table S4; Figureueras et al., 2014). Based on an ANI value of 100, the following co-isolated strains are likely to be clones but were kept for further analysis: ESM429_1 and ESM429_3; ESF442_5 and ESF442_6; and ESF436_3, ESF436_4, and ESF436_6.

The genome of one reference isolate, *Vibrio campbellii* KNH1, was also sequenced to confirm its species identification. This strain had previously been assigned as *Vibrio parahaemolyticus* based on 16S rRNA gene Sanger sequencing (Nyholm et al., 2000). The 16S rRNA gene often cannot provide enough genetic diversity to assign a species level identification in vibrios (Silvester et al., 2017), so an ANI comparison to other reference genomes was performed. An ANI cutoff of ≥95% revealed that the KNH1 strain should be assigned to the species *V. campbellii* (Supplementary Table S5).

### T6SS characterization of light organ symbionts

**Figure 1:**
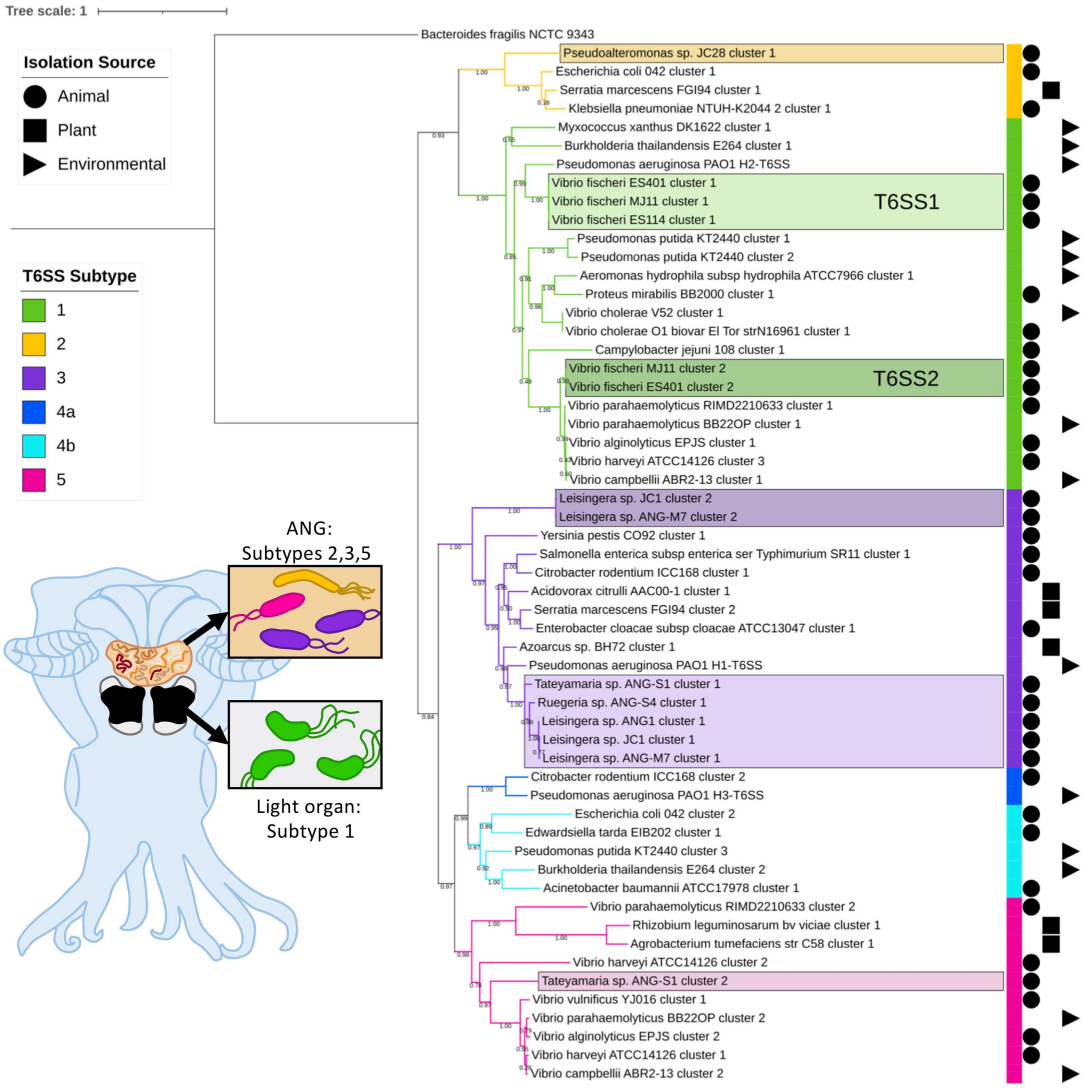
Maximum-likelihood phylogenetic tree of type VI secretion system (T6SS) sheath subunit proteins of *Euprymna scolopes* symbionts. A maximum-likelihood tree was constructed from the TssB and TssC small and large T6SS sheath subunit proteins predicted in select *Euprymna scolopes* accessory nidamental gland (ANG) and light organ bacterial symbiont genomes (names boxed) and reference genomes. The colored strip to the right of the tree and branch colors correspond to the T6SS subtype (Green = subtype 1, yellow = subtype 2, purple = subtype 3, dark blue = subtype 4a, light blue = subtype 4b, and pink = subtype 5). Symbols to the right of the colored strip indicate the isolation source of the sequenced bacterial strain (Circle = animal source, square = plant source, triangle = environmental source). Numbers on branches indicate bootstrap values. Inset cartoon diagram shows a ventral dissection of an adult female *E. scolopes* with colored ANG and light organ. Boxes show cartoon bacteria colored to represent the T6SS subtype of the symbionts found in each tissue type. See Supplementary Figure S1 for a complete phylogenetic tree with all strains analyzed in this study.

Of the 57 *V. fischeri* light organ symbiont genomes and 14 *V. fischeri* seawater isolate genomes analyzed, all had one predicted T6SS cluster on chromosome 1 (T6SS1) and 38 genomes had a second T6SS cluster on chromosome 2 (T6SS2; Figure 1; Supplementary Figure S1). Based on phylogenetic analysis of the sheath subunit protein sequences (TssBC), these clusters appear to share an evolutionary history, but have diversified to form two distinct clades within group one of the previously described T6SS subtypes (Boyer et al., 2009). The TssBC proteins of strains isolated from different animal hosts (*n* = 58) and seawater sources (*n* = 12) were observed to cluster together in both clades, indicating there is no evolutionary link between type of T6SS and isolation source (i.e., host-associated vs. environmental).

**Figure 2:**
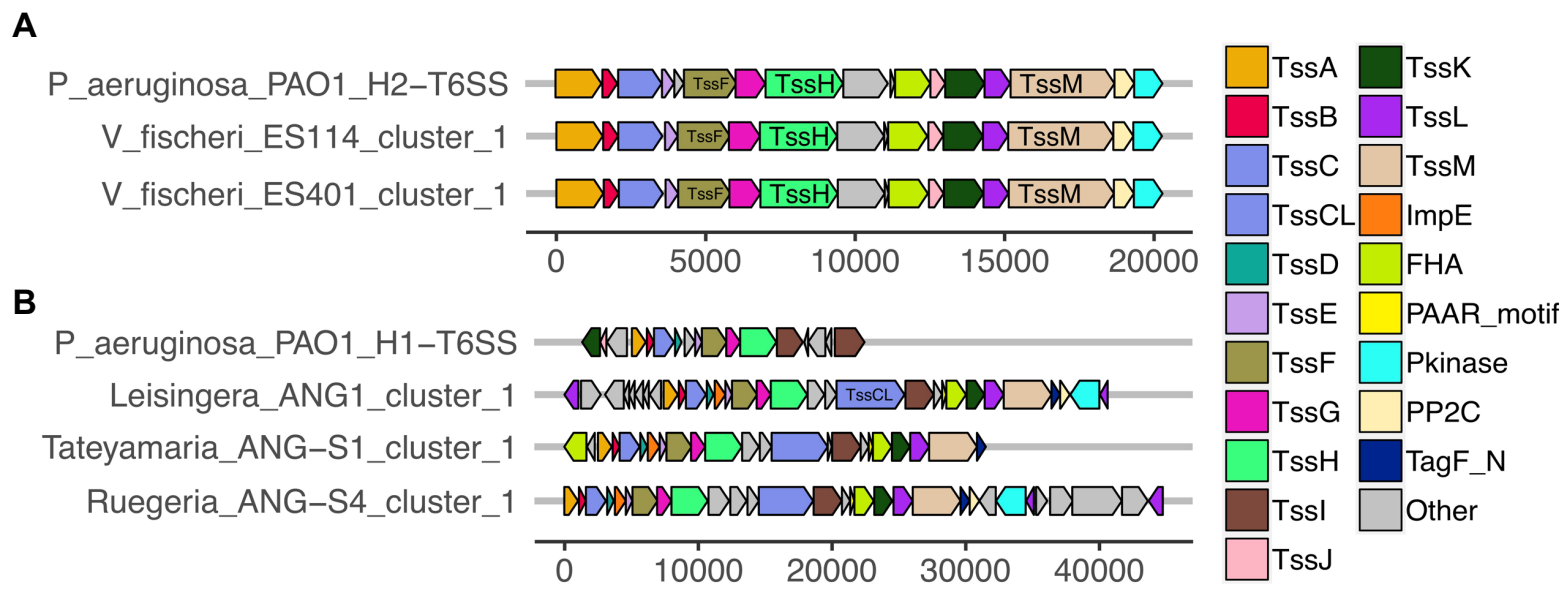
Predicted T6SS gene clusters of *Euprymna scolopes* symbiont genomes share similar gene organization to T6SS gene clusters in *Pseudomonas aeruginosa* PAO1. Each of the T6SS core structural and accessory genes are colored, non-T6SS genes are labeled as “other” and are gray. Genes are drawn to scale at the bottom of each cluster, measured in bp. **(A)** The T6SS1 of *Vibrio fischeri* genomes share similar gene content and organization to the H2-T6SS of *P. aeruginosa* PAO1. **(B)** The first T6SS clusters of ANG strains share similarity to the H1-T6SS of *P. aeruginosa* PAO1.

All of the predicted *V. fischeri* T6SSs share evolutionary similarity to other well characterized T6SS model systems. The *V. fischeri* T6SS1 TssBC proteins were most phylogenetically similar to the H2-T6SS of *Pseudomonas aeruginosa* PAO1, and both clusters share the same core T6SS gene organization (Figure 2A). The *V. fischeri* T6SS2 TssBC proteins are most similar to TssBC in several other vibrios, including *V. parahaemolyticus*, *V. alginolytiucs*, *V. harveyi,* and *V. campbellii*, as was previously seen in a search of *V. fischeri* IcmF (TssM) homologs (Speare et al., 2018).

All *V. fischeri* T6SS1 clusters contain 11 of the 13 core structural genes (*tssA*-*tssM*) but are missing *tssD* and *tssI*, which encode the Hcp tube and the VgrG spike proteins, respectively (Supplementary Table S3). These genes are often located in auxiliary clusters elsewhere in the genome (Dong et al., 2013; Speare et al., 2018), which were excluded from our analysis. The *V. fischeri* T6SS1 clusters also contain three accessory genes with protein phosphatase 2C (PP2C), protein kinase (Pkinase), and forkhead-associated (FHA) domains, which may be involved in post-translational regulation, as seen in *P. aeruginosa* PAO1 (Mougous et al., 2007).

Of the *V. fischeri* T6SS2 clusters, 79% (30/38) contain all the core *tssA*-*M* structural genes as well as six accessory genes (Supplementary Table S3). The remaining eight genomes with incomplete T6SS2 clusters are draft assemblies where the T6SS2 cluster was cutoff at the beginning or end of a contig (Supplementary Table S3). The break in the assemblies in this locus could account for the missing genes, which would go undetected by our analysis criteria. The accessory genes in T6SS2 also included pkinase, FHA, and DUF4150 domain-containing proteins similar to those found in T6SS1, as well as the recently described *tasL* gene, which is required for cell–cell adhesion and target specificity (Speare et al., 2022), and the *tasR* (Smith et al., 2021) and *vasH* (Guckes et al., 2020) regulators.

**Figure 3:**
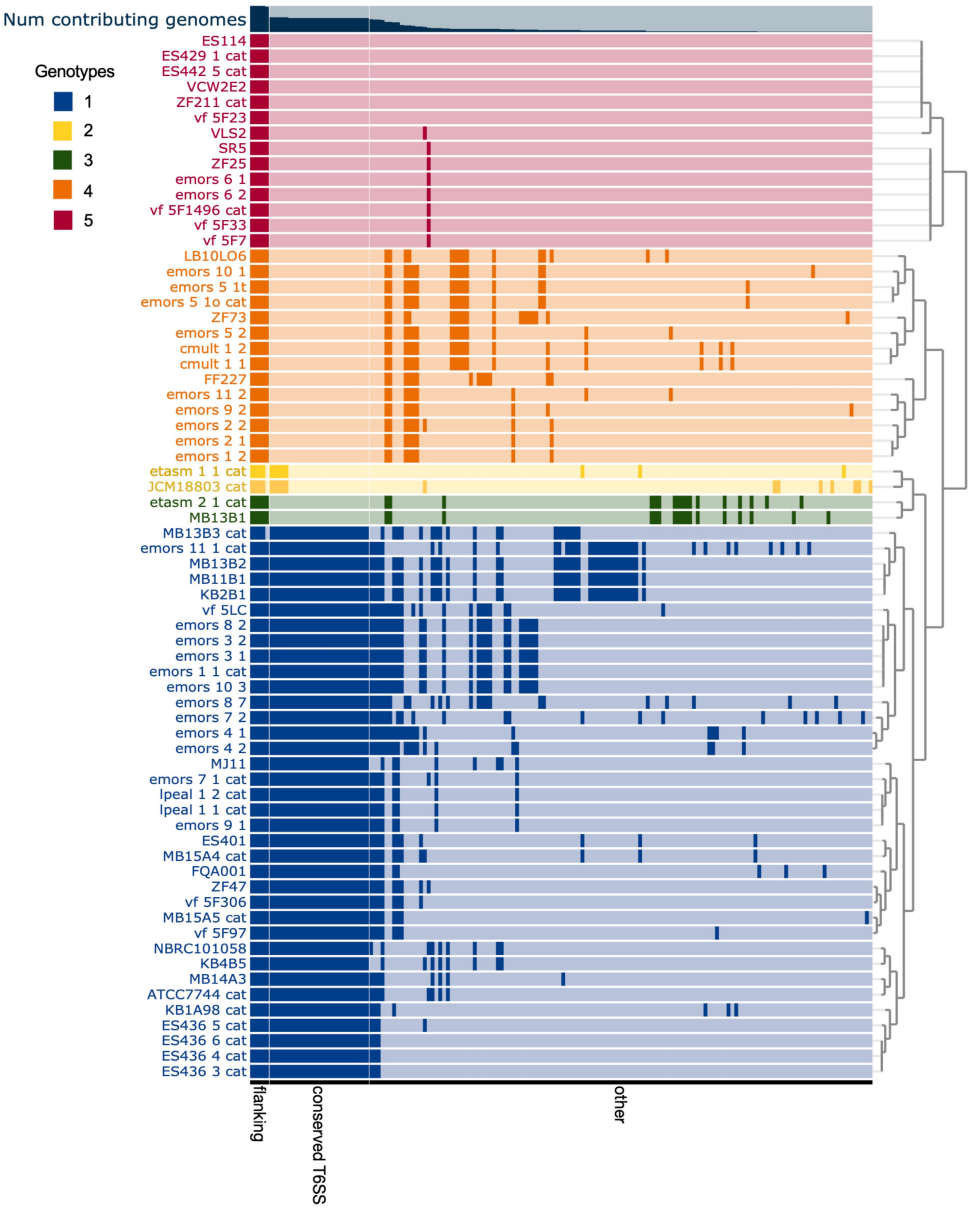
Comparison of predicted *Vibrio fischeri* T6SS2 genomic island regions reveals five distinct genotypes. Each row represents the T6SS2 genomic region of each *V. fischeri* strain based on presence of the *manA* and tRNA-Gly flanking genes. Gene clusters with shared similarity between strains are a darker shade of the row color. The strains are ordered based on hierarchical clustering of each T6SS2 genomic region, which revealed five distinct genotypes: (1, blue) a complete T6SS2 cluster and other genes, (2, yellow) an incomplete T6SS2 cluster and other genes, (3, green/ 4, orange) no T6SS2 cluster but several other genes, or (5, red) few to no genes in this region. Bar graph at top of chart indicates the percent of genomes that possess that gene cluster. Gene clusters are ordered based on binning of flanking genes (present in all genotypes), conserved T6SS genes (present in genotypes 1 and 2), and other non-T6SS genes which vary between the genotypes.

**Figure 4:**
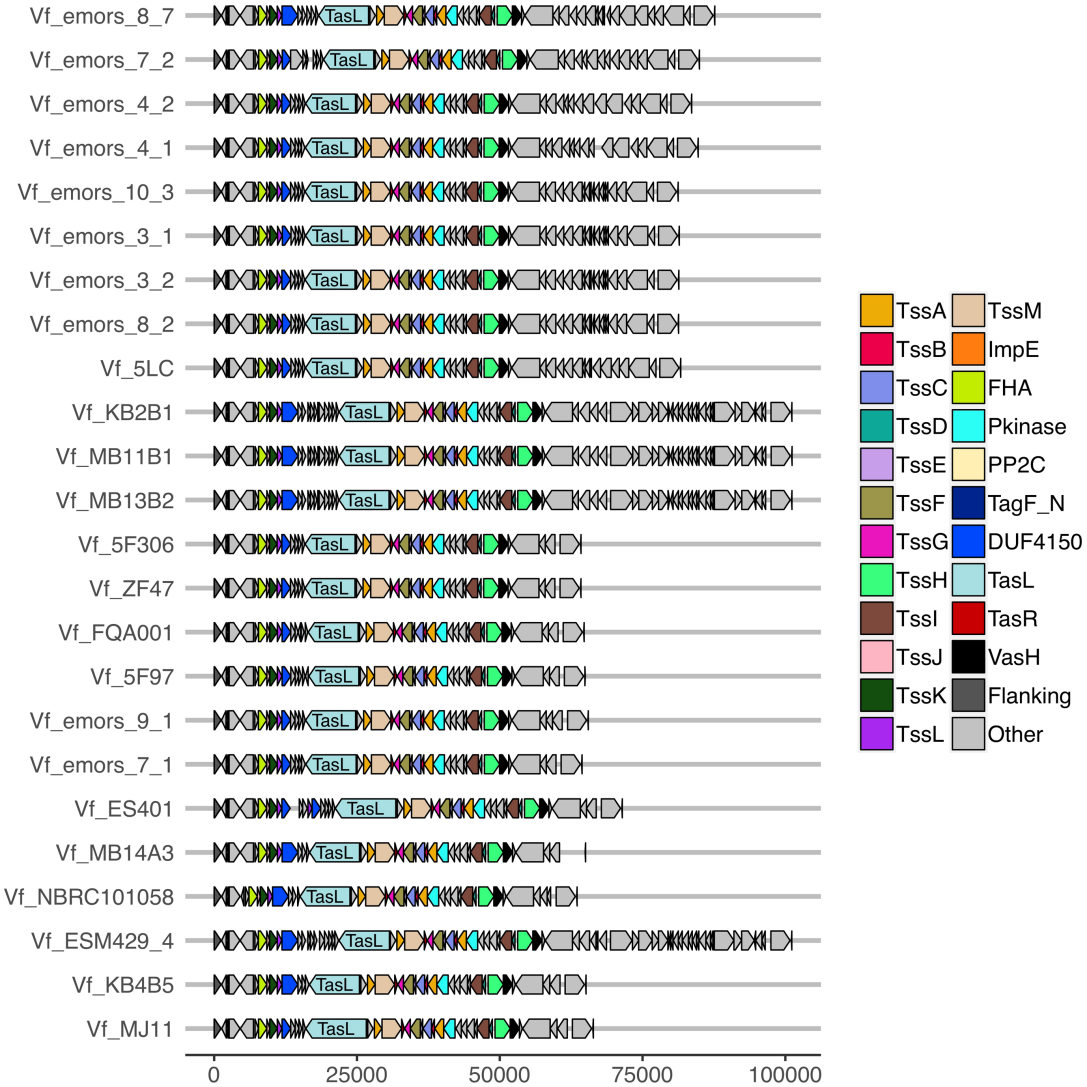
Predicted T6SS2 clusters of *Vibrio fischeri* genomes feature different patterns of cluster expansions. Gene organization of the predicted T6SS2 clusters of both fish and squid symbiont and environmental *V. fischeri* genomes. Genes labeled as “other” do not currently have a documented T6SS-associated function. See Supplementary Table S6 for a list of predicted annotations for proteins labeled as “other.” Genes are drawn to scale at bottom of figure, measured in bp.

The T6SS2 locus is within a genomic island with high variability among the examined *V. fischeri* genomes. Five distinct genotypes were identified in this region based on the presence of the flanking genes, *manA* and tRNA-glycine (Figure 3), with tRNAs often being a site of genomic island insertion (Darmon and Leach, 2014). The five genotypes in this region include genomes with (1) a complete T6SS2 cluster and other genes (52.9% of genomes), (2) an incomplete T6SS2 cluster and other genes (2.9% of genomes), (3/4) no T6SS2 cluster but several other genes (23.5% of genomes), or (5) few to no genes in this region (20.6% of genomes). The sizes of these different genotype regions, including the flanking genes, range from 11.2 to 106.2 kbp. Non-T6SS genes in this region included predicted type 1 restriction enzyme and CRISPR-associated proteins (Supplementary Table S6), which are defensive mechanisms that are often overrepresented in bacterial genomic islands (Merk, 2006). Within the first genomic island genotype (T6SS2+), there is a diversity of T6SS2 cluster expansions and accessory genes (Figure 4). The region between the PAAR-like DUF4150 containing gene and *tasL* varies from 3 to 11 genes depending on the strain, while the region after *vasH* varies from 4 to 31 genes. These regions encode possible effectors with PAAR-like domains, lysozyme-like domains, and other conserved domains of unknown functions, as well as restriction enzymes, nucleases, GTPases, ATPases, and OmpA proteins. This finding indicates that although auxiliary clusters containing effector/immunity pairs exist, the primary T6SS2 gene cluster is also capable of diversifying effector arsenals. Furthermore, there are still conserved genes of unknown function encoded within the *V. fischeri* T6SS2 gene cluster that remain to be characterized.

While a function for the *V. fischeri* T6SS1 has not yet been determined, the T6SS2 cluster has previously been shown to be required for intra- and interspecies competition in a variety of strains (see contact-dependent inhibition annotation on Supplementary Figure S1; Speare et al., 2018, 2020, 2022). We observed the same pattern in the nine *V. fischeri* isolates from *E. scolopes* light organs described in this study (Supplementary Table S2), where only strains that possess a T6SS2 cluster were able to kill *V. fischeri* ES114 in coincubation assays on agar plates (Supplementary Figures S1, S2). However, disruption of the T6SS2 would be required to verify if the ability to kill ES114 is due to this gene cluster.

### T6SS characterization of accessory nidamental gland symbionts

Of the 15 ANG strains analyzed, all but one (*Ruegeria* sp. ANG-R) had at least one predicted T6SS cluster (Figure 1; Supplementary Figure S1; Supplementary Table S3). All 11 *Leisingera* strains had at least one T6SS cluster that formed a clade in the group three T6SS subtype, along with T6SS clusters found in *Ruegeria* sp. ANG-S4 and *Tateyamaria* sp. ANG-S1. The TssBC proteins of these T6SS clusters were most phylogenetically similar to the H1-T6SS of *P. aeruginosa* PAO1, although the ANG strain clusters appear to have further expanded (Figure 2B). Two of the *Leisingera* strains, ANG-M7 and JC1, had a second T6SS cluster that formed a separate clade within group three. A second T6SS cluster identified in *Tateyamaria* sp. ANG-S1 was assigned to group five and was similar to T6SS clusters from several vibrios. The one T6SS cluster predicted for *Pseudoalteromonas* sp. JC28 fell within group two, along with *E. coli* and *Serratia marcescens* T6SS clusters.

**Figure 5:**
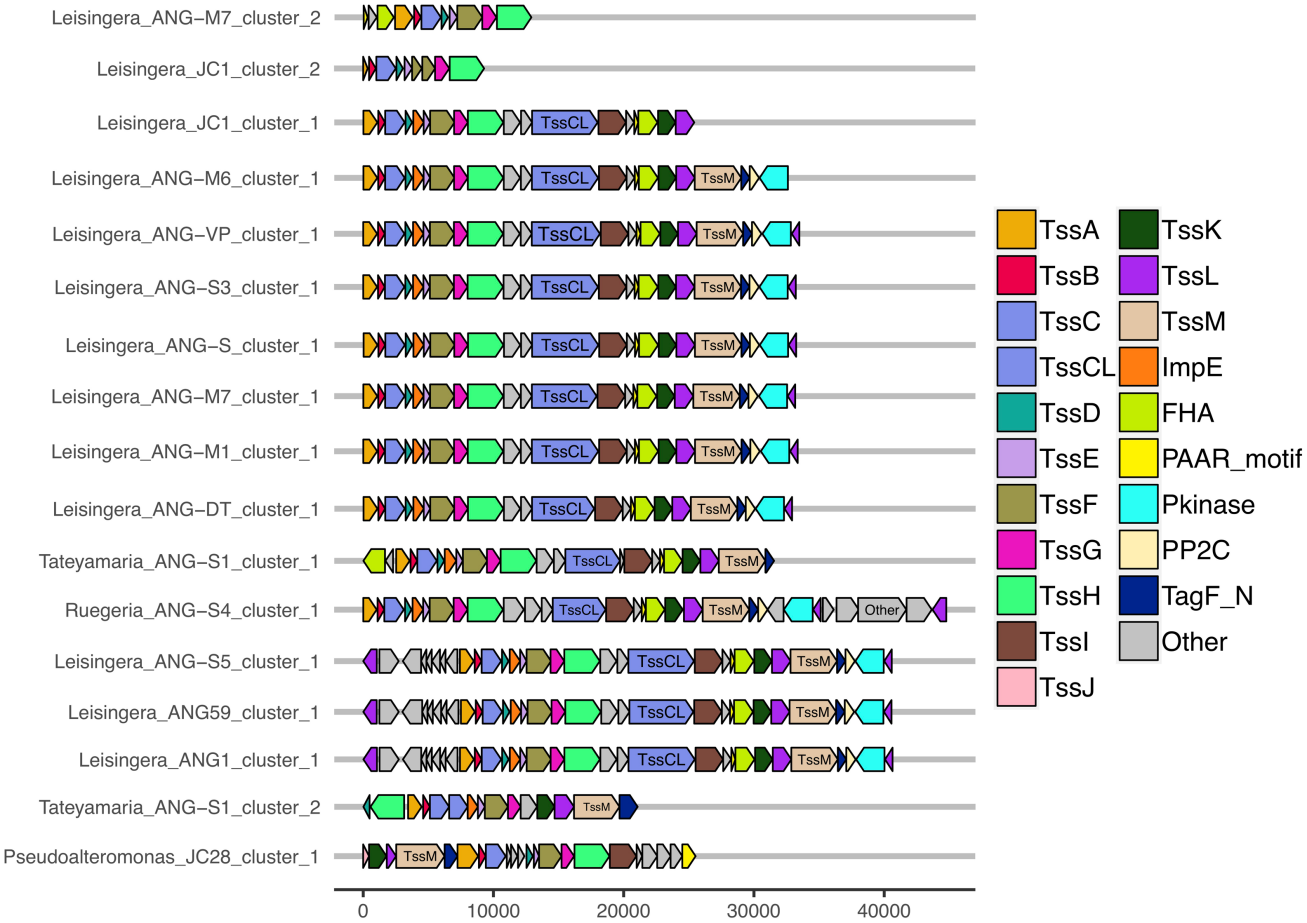
Gene organization of predicted T6SS clusters of *Euprymna scolopes* ANG bacterial symbionts. The roseobacter ANG symbiont genomes (all but *Pseudoalteromonas* sp. JC28) share similar T6SS accessory genes and cluster organization. These strains also share a second, long copy of the large sheath subunit, termed here TssC_L_, in their first T6SS clusters. TssC_L_ was not detected in any genomes outside of the *Alphaproteobacteria,* and is absent from the *Gammaproteobacteria* ANG symbiont, JC28, shown here. Each of the T6SS core structural and accessory genes are colored, genes that have not been associated with a T6SS function are labeled as “other” and are gray. Genes are drawn to scale at bottom of figure, measured in bp.

All of the roseobacter (*Leisingera*, *Ruegeria*, and *Tateyamaria*) T6SS1 clusters contained all of the core T6SS structural genes except *tssJ* (Supplementary Table S3), which encodes a protein that has been localized to the membrane complex in *Gammaproteobacteria* (Aschtgen et al., 2008). The operons for all of these clusters share similar gene organizations as well as the PAAR-motif, FHA-domain, and *impE* accessory genes (Figure 5). The *Pseudoalteromonas* sp. JC28 cluster contained all of the core T6SS structural genes along with the PAAR-motif gene and *tagF* regulatory gene. The second T6SS clusters of *Leisingera* spp. ANG-M7 and JC1 are smaller and may perform a different function or be non-functioning degenerate clusters (Figure 5). Cluster 2 of ANG-M7 contains eight of the 13 core genes (*tssA-H*) as well as two accessory genes (PAAR motif, FHA; Supplementary Table S3). Cluster 2 of JC1 also contains eight core genes (*tssA-H*) with a duplicate *tssF* gene and no accessory genes. Notably, both clusters lack core genes for assembling the membrane complex and VgrG spike protein (*tssI*). The second T6SS cluster of *Tateyamaria* sp. ANG-S1 is a more complete T6SS cluster, only missing the *tssI* and *tssJ* genes and could be functional (Supplementary Table S3).

**Figure 6:**
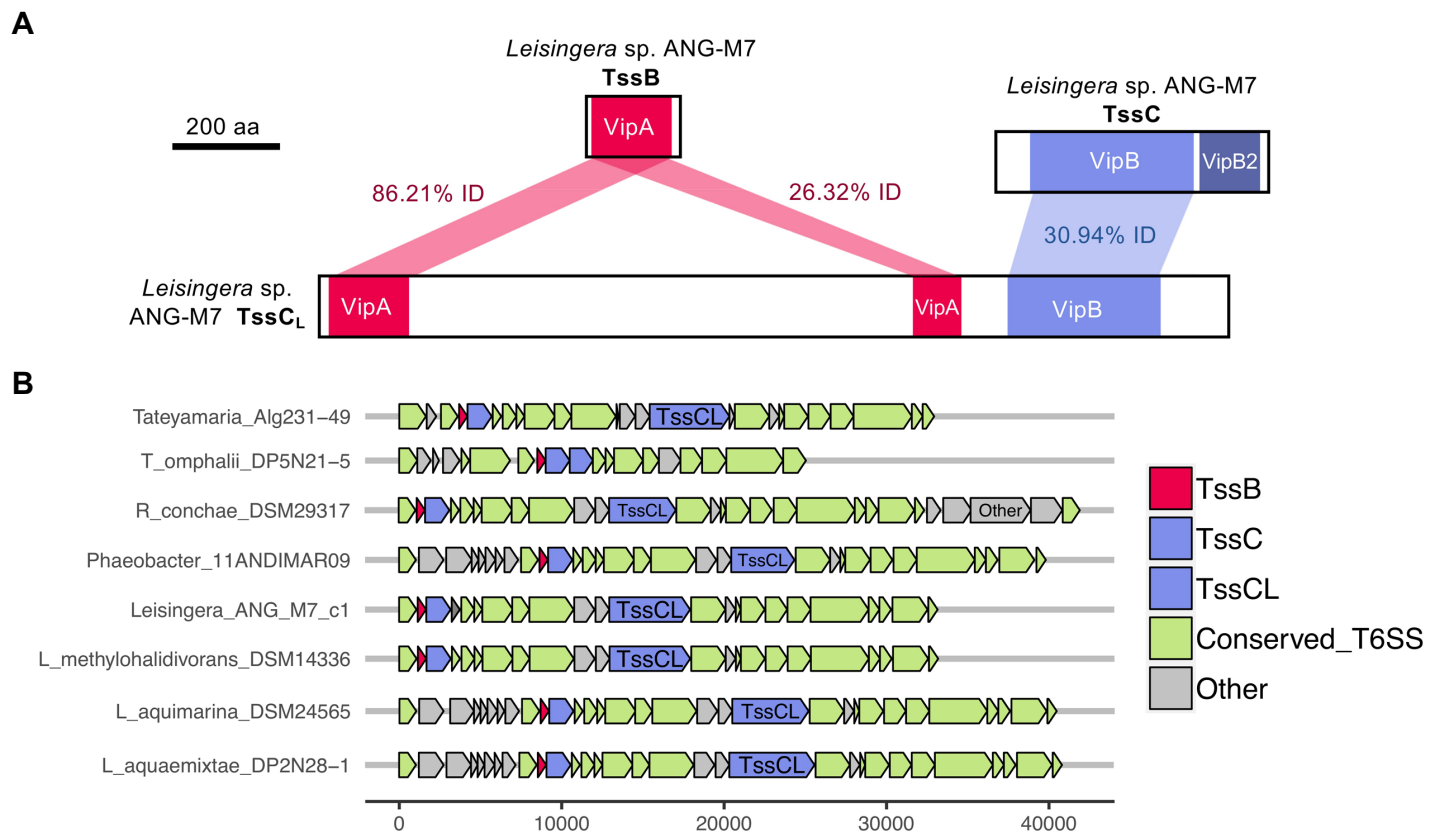
Domains and occurrence of the *Alphaproteobacteria* sheath protein, TssC_L_. The long copy of the TssC protein, termed here TssC_L_, contains domains from both TssB and TssC and was only detected in other roseobacter reference genomes in this study. **(A)** The TssC_L_ protein of *Euprymna scolopes* ANG symbiont *Leisingera* sp. ANG-M7 contains two VipA domains with 86.21% (1e^−94^ E-value) and 26.32% (5e^−5^ E-value) amino acid identity to the VipA domain in TssB. This TssC_L_ protein also has a VipB domain with 30.94% identity (2e^−36^ E-value) to the VipB domain in TssC. **(B)** Other roseobacter genomes that have a *tssC*_L_ gene still have *tssB* and *tssC* genes. Some roseobacter genomes, such as *Tateyamaria omphalii* DP5N21–5, do not have *tssC*_L_, but instead have two *tssC* genes and one *tssB* gene.

Interestingly, all of the T6SS1 clusters in the roseobacter ANG genomes contained two predicted *tssC* genes: one that encodes a protein of similar size to previously described TssC proteins (506 aa), and a second gene predicted to encode a much larger protein (1,684 aa; Figure 5). A pfam domain search of this longer TssC protein, which we term “TssC_L,_” revealed it contains two VipA domains (PF05591) and one VipB domain (PF05943; Figure 6A). Only one of the VipA domains shares high amino acid identity (86.2%) with the VipA domain in the TssB protein of the same T6SS cluster. The second VipA domain in TssC_L_ is a shorter sequence with only 26.3% amino acid identity to the same domain in TssB. The one VipB domain in TssC_L_ shared low amino acid identity (30.9%) to the first VipB domain in the shorter *tssC* gene. Although TssC_L_ is annotated as *tssC* in the rosoebacter genomes, it contains key domains from both sheath subunits and appears to be a fusion of the two genes.

Similar TssC_L_ proteins are present in other roseobacter T6SS clusters, but absent from all other bacterial genomes analyzed in this study. The TssC_L_ protein was found in 20 of the 28 roseobacter reference genomes with a similar predicted T6SS cluster, with amino acid identities to the ANG-M7 TssC_L_ ranging from 45 to 93% (Figure 6B; Supplementary Table S7). The domain architecture within TssC_L_ described above was also observed in seven other proteins in the pfam database, all from *Alphaproteobacteria*, that have 46–64% amino acid identity to the ANG-M7 TssC_L_ (Supplementary Table S7). Interestingly, four of the *Tateyamaria* T6SS clusters had two copies of *tssC* but no *tssC_L_* (Figure 6B). Within each cluster, the two TssC proteins had 34–37% amino acid identity to each other (Supplementary Table S7), suggesting they did not arise from a gene duplication event. Thus, it is possible that the TssC_L_ protein may have arisen from a fusion of multiple tail sheath subunits genes. Moreover, the *tssC_L_* gene is absent in the closely related H1-T6SS gene cluster of *P. aeruginosa* PAO1 (Figure 2), suggesting it is a more recent evolutionary event that occurred in certain *Alphaproteobacteria*.

As was seen for the *V. fischeri* genomes, there is no evidence to suggest that T6SS gene clusters are enriched in the host-associated isolation sources, relative to free-living environmental sources (Supplementary Figure S1; Supplementary Table S3). All 11 of the *Leisingera* reference genomes present in Genbank and analyzed in this study were from environmental sources and also possessed a T6SS cluster. For comparison, other roseobacter genomes were also analyzed and only three of 55 *Phaeobacter* reference genomes and five of 31 *Ruegeria* reference genomes possess a T6SS (Supplementary Table S3), thus possession of the T6SS may be a conserved trait within the *Leisingera* genus. For the other genera represented in ANG strains, four of 15 *Tateyamaria* reference genomes and 14 of 15 *Pseudoalteromonas* reference genomes also possess a T6SS (Supplementary Table S3). Overall, although T6SS gene clusters are present in many host-associated strains, it was not determined to be enriched in the host environment, suggesting there may be a functional role for T6SS in both host- and free-living habitats.

To date, there are no described functions for the T6SS in any of the ANG symbionts, nor for any of the T6SSs of reference roseobacter genomes. To begin to understand what activity the ANG T6SS clusters may have, an effector search was completed for *Leisingera* sp. ANG-M7, a representative genome from the most dominant genera in the ANG symbiotic community (Collins et al., 2012; Kerwin and Nyholm, 2017). By alignment to a database of 326 experimentally validated effector protein sequences (SecReT6, Li et al., 2015), ANG-M7 had 39 significant predicted effector hits (Supplementary Table S8). Of these, four hits were to proteins in the T6SS cluster 1 (PAAR-motif, VgrG, Hcp, and a peptidase) and three to proteins in the T6SS cluster 2 (PAAR-motif, Hcp, and TssB). Two other VgrG and one PAAR-motif protein were found at other locations in the genome, as well as three amidases. The remaining significant hits included several cold shock proteins, LysR family transcriptional regulators, a phosphatidylserine synthase, a universal stress protein, and a tRNA ligase (Supplementary Table S8). While some of these predicted effector proteins may have similar domains to those in previously described T6SS effectors, it remains to be seen if they are indeed secreted through the T6SS in *Leisingera* sp. ANG-M7, and whether they are used during interbacterial competition and/or interactions with co-occurring eukaryotic cells.

## Discussion

In this study, we sought to characterize the type and abundance of T6SS gene clusters in symbionts of *E. scolopes* and found that all isolates from both the light organ and ANG, except one, possess at least one predicted T6SS cluster. Consistent with previous work by Speare et al., we observed that approximately half of *V. fischeri* strains isolated from wild-caught squid light organs possess a T6SS2 cluster, which was previously shown to be important for competition in the light organ crypts (Speare et al., 2018, 2022). Previously published phylogenies showed that *V. fischeri* T6SS2+ and T6SS2− strains are found dispersed among closely related strains, and do not cluster based on T6SS2 genotype (Speare et al., 2018), suggesting that there was an evolutionary loss of T6SS2. Here, we show that for some T6SS2− strains, different gene clusters can be found in place of T6SS2. Overall, *E. scolopes* symbionts possess T6SS clusters that represent four of the five described T6SS subtypes, with light organ symbiont T6SSs forming two clades in group 1 and the ANG symbiont T6SSs forming two clades in group 3 and one clade each in groups 2 and 5. Three of the roseobacter ANG symbionts possess a second T6SS cluster that may have been acquired from other bacteria based on their phylogenetic distance from the main roseobacter group 3 clade.

The Hawaiian bobtail squid, *E. scolopes*, is a good model system to study the interactions of bacterial strains possessing different T6SS subtypes during colonization of a single host. The diversity of T6SS subtypes detected in *E. scolopes* symbionts is expected due to the diversity of bacterial taxa represented, including a range of *Alphaproteobacteria* and *Gammaproteobacteria*. While the light organ only contains *V. fischeri* symbionts, the ANG is a more diverse community with only 17 isolate genomes available in Genbank at this time. Our reported diversity of T6SS clusters in the ANG is likely an underestimate because our study was limited to the cultured isolate genomes, which only represent a few genera of the community based on 16S rRNA gene sequencing (Collins et al., 2012; Kerwin and Nyholm, 2017). Despite this limitation, we still observed representatives of three T6SS subtypes within the ANG alone. Future research will determine if any of these T6SS clusters, besides *V. fischeri* T6SS2, are expressed and form sheathes in the host and what role they might play during symbiosis.

While the functional activity of ANG symbiont T6SSs remains to be determined, potential effectors were identified in one strain that could suggest their ability to inhibit other bacteria through contact-dependent competition. It has been previously shown through fluorescence *in situ* hybridization that different bacterial taxa in the ANG are observed in separate tubules (Collins et al., 2012). It has also been seen that the relative abundance of different bacterial classes shift as the ANG develops, which occurs as the nascent gland tissue forms pores that lead to invaginations that will become tubules in the mature gland (Kerwin et al., 2021). Similar to the light organ crypts, where T6SS2-dependent competition shapes the diversity and spatial arrangement of *V. fischeri* strains, the ANG tubules would provide a similar niche for ANG symbiont competition during colonization. The observed community shifts in the ANG could be attributed, at least in part, to contact-dependent inhibition mechanisms like the T6SS being deployed in initially co-colonized tubules. Future research will determine whether ANG symbionts are actively using their T6SSs to compete in the host as has been previously seen for light organ symbionts (Speare et al., 2018, 2022).

The phylogenetic analysis based on the structural TssBC proteins found that T6SS clusters from *E. scolopes* symbionts are similar to T6SSs in other well-studied model systems with impacts on animal hosts. In particular, the T6SS1 of light organ symbionts were most similar to the *P. aeruginosa* PAO1 H2-T6SS and the T6SS cluster 1 of roseobacter ANG symbionts was most similar to *P. aeruginosa* PAO1 H1-T6SS. The H1-T6SS of *P. aeruginosa* PAO1 can secrete three effector proteins, Tse1–3, which can target peptidoglycan and arrest bacterial cell growth (Hood et al., 2010; Russell et al., 2011). The Hcp of H1-T6SS has also been detected in the pulmonary secretions of cystic fibrosis patients (Mougous et al., 2006) and has higher expression in biofilms compared to in planktonic cells (Zhang et al., 2011). The H2-T6SS of *P. aeruginosa* PAO1 is regulated by quorum sensing and iron-limiting conditions and contributes to internalization by human cell lines and virulence in *C. elegans* (Sana et al., 2012). Although these studies associated PAO1’s H2-T6SS with effectors that impact eukaryotic cells, the phospholipase effector, PldA, secreted by this cluster has been shown to have antibacterial activity by degrading phosphatidylethanolamine of bacterial cell membranes (Russell et al., 2013). Thus, both H1-T6SS and H2-T6SS of PAO1 can contribute to effects on animal hosts and interbacterial competition. While similarity exists between the structural components of *P. aeruginosa* PAO1 T6SS clusters and *E. scolopes* symbiont T6SS clusters, it does not necessarily suggest that these clusters will have similar functions. These studies in PAO1 and other T6SS models have shown that the impact of a T6SS is governed by its effectors (Russell et al., 2014a). While initial investigation of *Leisingera* sp. ANG-M7’s effectors, and previous evidence in *V. fischeri* T6SS2 activity (Speare et al., 2018), point to antibacterial functions, it remains to be seen what alternative roles these or remaining uncharacterized effectors might play in the *E. scolopes* symbioses.

Finally, this study identified a new T6SS structural protein, TssC_L_, among certain roseobacter genomes that appears to be a fusion of two broadly conserved sheath subunits, TssB and TssC. The TssB and TssC small and large sheath subunits form heterodimers that stack on top of each other and contract during firing to deliver effectors (Costa et al., 2015). In other systems, fusion proteins have been detected from protein subunits that directly interact, such as domains of GyrA and GyrB in *E. coli* that are found in the topoisomerase II fusion protein in yeast (Enright et al., 1999; Marcotte et al., 1999). Thus, due to their dimer formation, TssB and TssC sheath subunits are likely candidates for fusion. However, this is not the first instance of apparent fusion between T6SS proteins. In such bacteria as *Agrobacterium tumefaciens, Nitrococcus mobilis,* and *Rhizobium leguminosarum*, the *tagF* and *pppA* orthologs are fused (Silverman et al., 2011), but these genomes do not continue to carry single copies of the unfused genes. Thus, it will be important to determine the extent to which TssC_L_ and the single-copy TssB and TssC proteins interact to form a functional sheath in roseobacters.

The prevalence of T6SS clusters in *E. scolopes* symbionts suggests that possession of this interbacterial weapon might be beneficial during at least one life stage of the symbionts. Light organ symbionts are horizontally acquired from the water column after the squid hatch (Wei and Young, 1989), and it is believed that the ANG symbionts are also horizontally acquired when females begin to sexually mature (Kerwin and Nyholm, 2017). Thus, these symbionts must be equipped to survive in both free-living and host-associated conditions. Indeed, previous research in our lab has demonstrated that the *V. fischeri* T6SS2 is necessary for strain competition during light organ colonization (Speare et al., 2018, 2022). In other beneficial roles, the T6SS can also play a part in competitive exclusion, such as in the human gut (Russell et al., 2014b) and plant leaves (Bernal et al., 2017), and controlling host range in *Rhizobium* root nodulation (Bladergroen et al., 2003). More research on the role of T6SSs in beneficial symbioses is needed, and *Euprymna scolopes* will be a valuable model system to understand these roles in a diversity of beneficial symbionts and host-colonization sites.

## Data availability statement

The datasets presented in this study can be found in online repositories. The names of the repository/repositories and accession number(s) can be found in the article/Supplementary material.

## Author contributions

AMS, ANS, SS, and LS conceptualized and designed the study. AMS, SS, LS, YC, IC, EC, MK, AW, HW, and ANS isolated strains used in this study and conducted experiments. AMS, SS, YC, and ANS performed bioinformatic analyses. AMS and ANS wrote the manuscript. All authors contributed to the article and approved the submitted version.

## Funding

Research was funded by the UNC-CH Undergraduate Research Consultant Team program and NIGMS grant R35 GM137886 to ANS. AMS was supported by a NIGMS IRACDA postdoctoral fellowship at UNC-CH. SS was supported by a National Defense Science and Engineering Graduate Fellowship. LS was supported by a UNC Dissertation Completion Fellowship.

## Conflict of interest

The authors declare that the research was conducted in the absence of any commercial or financial relationships that could be construed as a potential conflict of interest.

## Publisher’s note

All claims expressed in this article are solely those of the authors and do not necessarily represent those of their affiliated organizations, or those of the publisher, the editors and the reviewers. Any product that may be evaluated in this article, or claim that may be made by its manufacturer, is not guaranteed or endorsed by the publisher.

## Supplementary material

SUPPLEMENTARY FIGURE S1 Expanded maximum-likelihood phylogenetic tree of T6SS sheath subunit proteins of *Euprymna scolopes* symbionts. A maximum-likelihood tree was constructed from the TssB and TssC small and large T6SS sheath subunit proteins predicted in all *E. scolopes* ANG and light organ bacterial symbiont genomes (names boxed) and reference genomes analyzed in this study. The colored strip to the right of the tree and branch colors correspond to the T6SS subtype (Green = subtype 1, yellow = subtype 2, purple = subtype 3, dark blue = subtype 4a, light blue = subtype 4b, pink = subtype 5). Symbols next to the colored strip indicate the isolation source of the sequenced bacterial strain (Circle = animal source, square = plant source, triangle = environmental source). Stars next to *V. fischeri* strain names indicate if a strain has been experimentally verified to be lethal against *V. fishceri* ES114 in a contact-dependent inhibition assay (colored star = lethal, white star = non-lethal). Numbers on branches indicate bootstrap values.

SUPPLEMENTARY FIGURE S2 Coincubation assay confirms that *V. fischeri* strains with a predicted T6SS2 gene cluster are also lethal against *V. fischeri* ES114. *V. fischeri* light organ symbionts isolated in this study were coincubated in mixed spots with the GFP tagged *V. fischeri* ES114 pVSV102. Strains that inhibit growth of *V. fischeri* ES114 do not show any GFP fluorescence in imaged colonies. Strains that allow growth of *V. fischeri* ES114 show GFP fluorescence in imaged colonies. Representative images are shown from three experimental trials, where each trial contained three resuspensions per strain, prepared from different colonies.
