# Supplementary figures and images for: Prevalence and diversity of type VI secretion systems in a model beneficial symbiosis
Source: Front Microbiol. 2022 Sep 14;13:988044. doi: 10.3389/fmicb.2022.988044 (PMC9515649; doi:10.3389/fmicb.2022.988044)

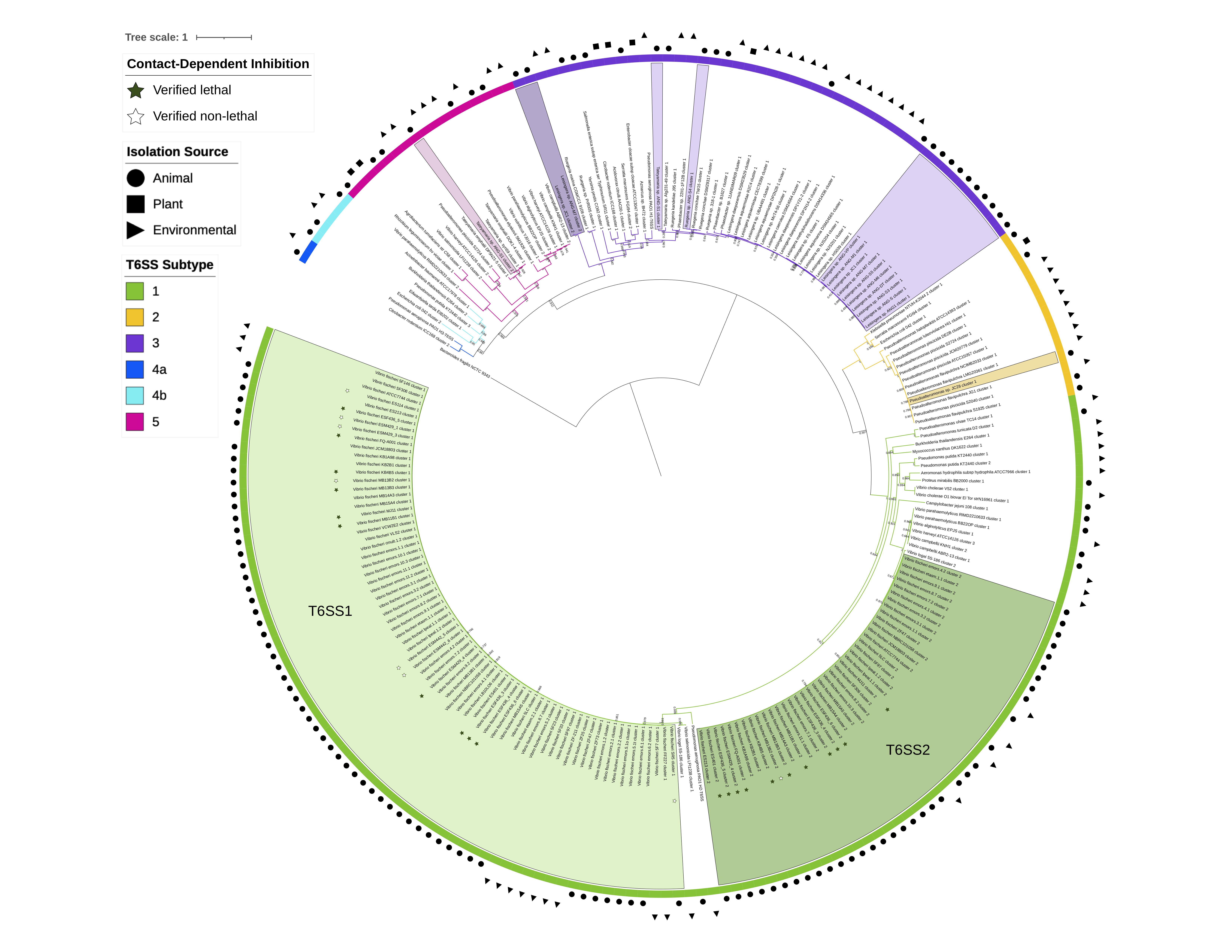

Supplement: SUPPLEMENTARY FIGURE S1 — Expanded maximum-likelihood phylogenetic tree of T6SS sheath subunit proteins of Euprymna scolopes symbionts. A maximum-likelihood tree was constructed from the TssB and TssC small and large T6SS sheath subunit proteins predicted in all E. scolopes ANG and light organ bacterial symbiont genomes (names boxed) and reference genomes analyzed in this study. The colored strip to the right of the tree and branch colors correspond to the T6SS subtype (Green = subtype 1, yellow = subtype 2, purple = subtype 3, dark blue = subtype 4a, light blue = subtype 4b, pink = subtype 5). Symbols next to the colored strip indicate the isolation source of the sequenced bacterial strain (Circle = animal source, square = plant source, triangle = environmental source). Stars next to V. fischeri strain names indicate if a strain has been experimentally verified to be lethal against V. fishceri ES114 in a contact-dependent inhibition assay (colored star = lethal, white star = non-lethal). Numbers on branches indicate bootstrap values. [file Image_1.JPEG]

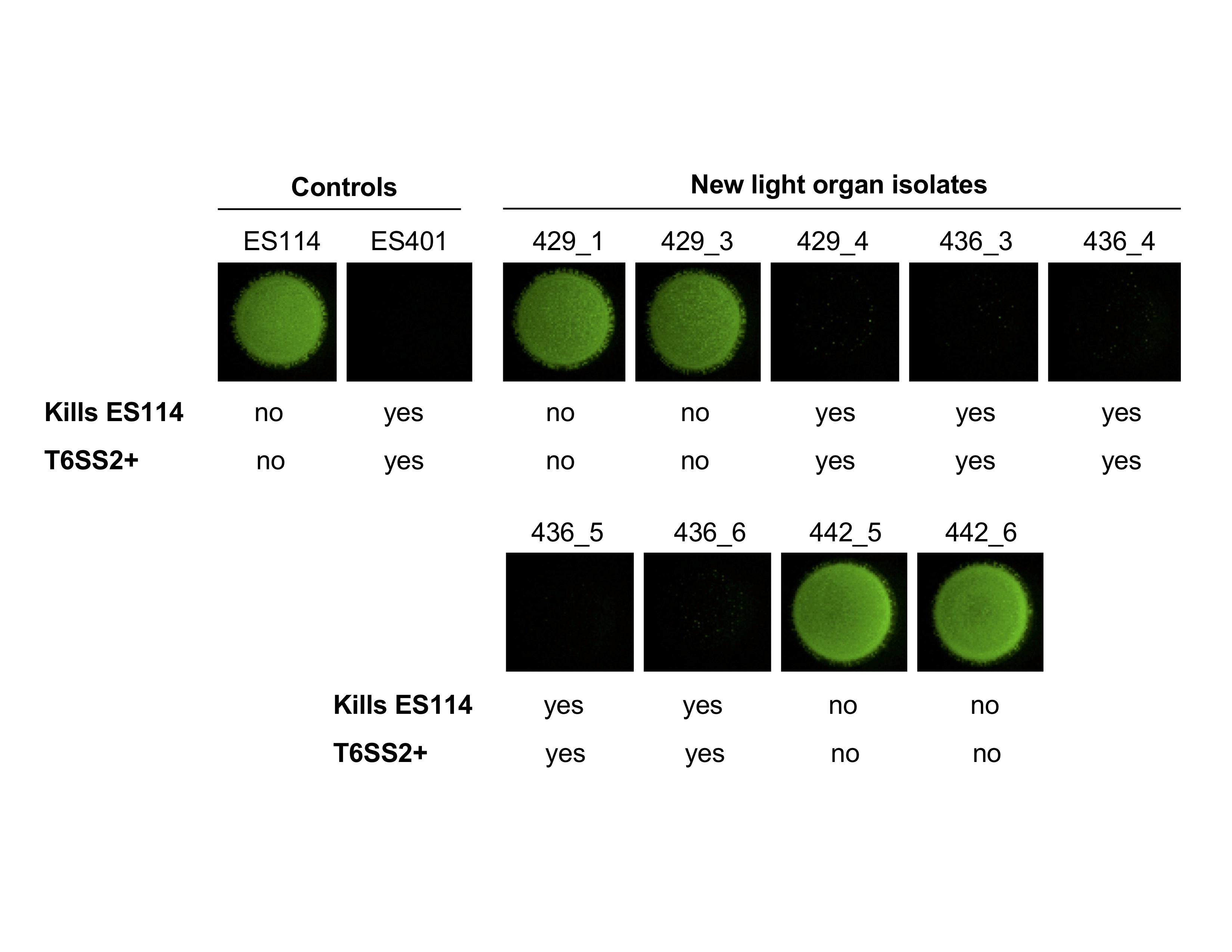

Supplement: SUPPLEMENTARY FIGURE S2 — Coincubation assay confirms that V. fischeri strains with a predicted T6SS2 gene cluster are also lethal against V. fischeri ES114. V. fischeri light organ symbionts isolated in this study were coincubated in mixed spots with the GFP tagged V. fischeri ES114 pVSV102. Strains that inhibit growth of V. fischeri ES114 do not show any GFP fluorescence in imaged colonies. Strains that allow growth of V. fischeri ES114 show GFP fluorescence in imaged colonies. Representative images are shown from three experimental trials, where each trial contained three resuspensions per strain, prepared from different colonies. [file Image_2.JPEG]
